# IDEAL-IQ in an oncologic population: meeting the challenge of concomitant liver fat and liver iron

**DOI:** 10.1186/s40644-018-0167-3

**Published:** 2018-12-12

**Authors:** Sarah Eskreis-Winkler, Giuseppe Corrias, Serena Monti, Junting Zheng, Marinela Capanu, Simone Krebs, Maggie Fung, Scott Reeder, Lorenzo Mannelli

**Affiliations:** 1 Department of Radiology, Memorial Sloan Kettering Cancer Center, 1275 York Avenue, New York, NY 10065 USA; 2 Department of Radiology, University of Cagliari, Via Università, 40, 09124 Cagliari, CA Italy; 3 Department of Statistics, Memorial Sloan Kettering Cancer Center, 1275 York Avenue, New York, NY 10065 USA; 4 IRCCS SDN, Naples, Italy; 5 Global MR Applications and Workflow, GE Healthcare, New York, NY USA; 6 Department of Radiology, University of Wisconsin-Madison, Madison, WI USA; 7 300 East 66th Street, New York, NY 10021 USA

**Keywords:** Fat fraction, Liver, PDFF, IDEAL-IQ, Chemotherapy, Oncologic imaging

## Abstract

**Background:**

Cancer patients often have a history of chemotherapy, putting them at increased risk of liver toxicity and pancytopenia, leading to elevated liver fat and elevated liver iron respectively. T1-in-and-out-of-phase, the conventional MR technique for liver fat assessment, fails to detect elevated liver fat in the presence of concomitantly elevated liver iron. IDEAL-IQ is a more recently introduced MR fat quantification method that corrects for multiple confounding factors, including elevated liver iron.

**Methods:**

This retrospective study was approved by the institutional review board with a waiver for informed consent. We reviewed the MRI studies of 50 cancer patients (30 males, 20 females, 50–78 years old) whose exams included (1) T1-in-and-out-of-phase, (2) IDEAL-IQ, and (3) T2* mapping. Two readers independently assessed fat and iron content from conventional and IDEAL-IQ MR methods. Intraclass correlation coefficient (ICC) was estimated to evaluate agreement between conventional MRI and IDEAL-IQ in measuring R2* level (a surrogate for iron level), and in measuring fat level. Agreement between the two readers was also assessed. Wilcoxon signed rank test was employed to compare iron level and fat fraction between conventional MRI and IDEAL-IQ.

**Results:**

Twenty percent of patients had both elevated liver iron and moderate/severe hepatic steatosis. Across all patients, there was high agreement between readers for IDEAL-IQ fat fraction (ICC = 0.957) and IDEAL R2* (ICC = 0.971) measurements, but lower agreement for conventional fat fraction measurements (ICC = 0.626). The fat fractions calculated with IOP were statistically significantly different from those calculated with IDEAL-IQ (reader 1: *p* < 0.001, reader 2: *p* < 0.001).

**Conclusion:**

Fat measurements using IDEAL-IQ and IOP diverged in patients with concomitantly elevated liver fat and liver iron. Given prior work validating IDEAL-IQ, these diverging measurements indicate that IOP is inadequate to screen for hepatic steatosis in our cancer population.

## Background

Patients at tertiary care cancer centers often suffer from disseminated disease and are treated with chemotherapy regimens [1–3]. Chemotherapy can lead to a wide range of adverse effects, including hepatoxicity and pancytopenia. An early sign of hepatotoxicity is hepatic steatosis, which can be recognized on medical imaging studies by indirect signs of the accumulation of fat globules within hepatocytes [4, 5]. Detecting hepatic steatosis is clinically important, as it often triggers changes in a patient’s medical and/or surgical treatment plans [6, 7].

Chemotherapy patients are also at risk for pancytopenia, which occurs as a result of chemotherapy-induced bone marrow failure. This often necessitates repeat blood transfusions, which can cause elevated liver iron levels. As such, this patient population may have a higher incidence of concomitantly elevated liver fat and elevated liver iron. It is therefore particularly important that the Magnetic Resonance Imaging (MRI) methods used for liver fat detection in this patient population not be confounded by elevated liver iron [8–11].

T1-in-and-out-of-phase (IOP) MRI is the conventional MR method for determining hepatic steatosis, and is included in all abdominal MR protocols at most institutions. In the IOP method, an initial “out-of-phase” (OP) set of images is acquired when there is destructive inference of the water and fat signals, and a subsequent “in phase” (IP) set of is acquired when there is constructive interference of the water and fat signals. The OP and IP images are then used to qualitatively measure liver fat fraction using the following eq. (SI = signal intensity) [12]:

$$ Liver\  fat\  fraction\%=\frac{\frac{SI\ {InPhase}_{liver}}{SI\ {InPhase}_{spleen}}-\frac{SI\ {OutofPhase}_{liver}}{SI\ {OutofPhase}_{spleen}}}{2\ast \frac{SI\ {InPhase}_{liver}}{SI\ {InPhase}_{spleen}}} $$

However, in patients containing both elevated liver iron and elevated liver fat, fat-induced and iron-induced signal changes cancel, leading to underestimation of both liver fat and liver iron content [11, 13]. In our patient population, where there may be a high incidence of concomitant elevated liver iron and elevated liver fat, a more robust MR method is needed [14].

Iterative Decomposition of water and fat with Echo Asymmetry and Least squares estimation (IDEAL-IQ) is a more sophisticated chemical-shift encoded fat quantification approach that corrects for several confounding factors, including T1 bias, eddy currents, noise bias, and T2* effects [9, 15, 16]. In IDEAL-IQ, images are acquired at multiple echo times, and an iterative least-squares decomposition algorithm is employed to simultaneous solve for a fat fraction map, a water fraction map, and an R2* map. By incorporating an R2* map into the algorithm, IDEAL-IQ accounts for T2* effects/field inhomogeneity, and yields a proton density fat fraction (PDFF) not confounded by iron overload [17–21]. IDEAL-IQ also outputs an R2* map, which can be used to identify the presence of iron overload. IDEAL-IQ has been validated in phantoms, in animal models, and in patients, with pathological gold standards [22–27]. Specifically, it has been shown to be highly reliable in patients with iron overload.

However, the utility of IDEAL-IQ has not yet been assessed in an oncologic population. At cancer centers, we expect higher rates of patients with concomitantly elevated liver fat and liver iron. And so, in this paper, we assess the utility of IDEAL-IQ by evaluating to what extent IDEAL-IQ liver fat and liver iron measurements diverge from measurements obtained with conventional IOP and T2* mapping. To accomplish this task, we evaluate agreement between IDEAL-IQ and IOP methods, as well as the inter-reader agreement of these methods.

## Methods

### Study population

This retrospective study was performed after obtaining an institutional review board-approved waiver of informed consent. Of all patients that received a liver MRI at our institution between May 2016 and June 2017 and who underwent at least 1 cycle of chemotherapy, 54 consecutive patients were identified whose MRI studies included: [1] T1-in-and-out-of-phase, [2] IDEAL-IQ and [3] T2* mapping. Patient age ranged from 5 to 76 (mean = 36.7). There were 32 males and 22 females.

### MRI imaging parameters

All patients were scanned on a 1.5 T MRI (OptimaMR450w, GE Healthcare) with a 32 Channel Torso Array Coil. The IDEAL IQ sequence had the following parameters: TR 10; TE 4.7; number of echoes 6, ranging from 1.1 ms to 6.38 ms FOV 35–40 cm; matrix size 128 × 128; pixel bandwidth 325 Hz; flip angle 6; slice thickness 10 mm; space between slices 5 mm. The scan was acquired during a single breath hold, lasting less than 30 s. The IOP sequence had the following parameters: single breath hold acquisition lasting 15 s, TR: 150 ms, out-of-phase TE 2.115 ms, in-phase TE: 4.252, FOV: 35–40 cm, matrix 320 × 192, slice thickness 7 mm, space between slices 1 mm.

Post-processing software, provided by the manufacturer, was used to generate fat fraction maps and T2*/R2* maps [28].

### Image post-processing

**Fig. 1:**
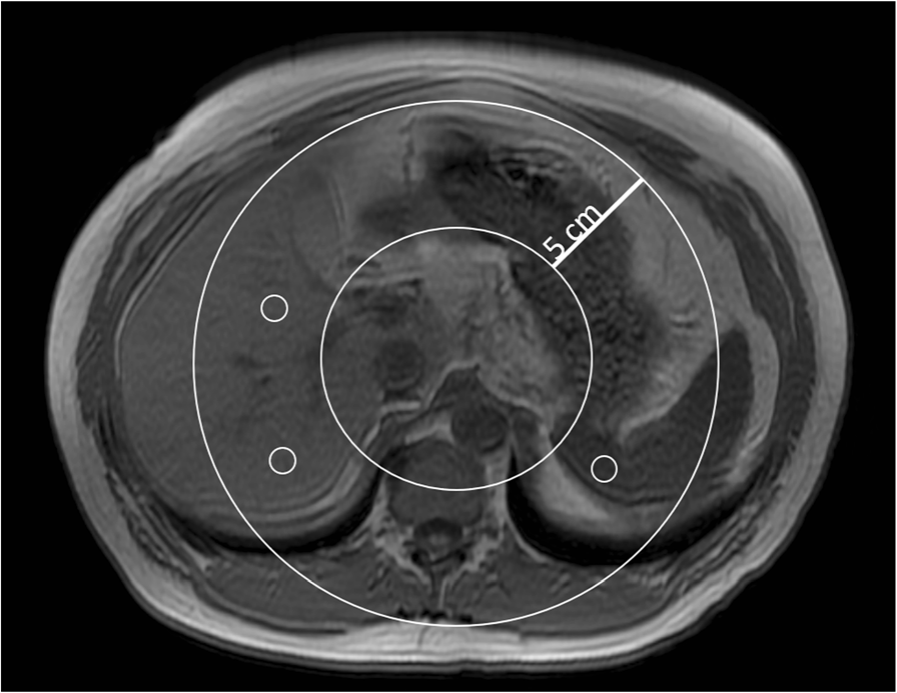
ROI placement. A 5 cm thick band was placed around the bore isocenter. Readers were instructed to place 3 ROIs in the liver and 3 in the spleen, on a plane passing through the portal vein division. Readers were instructed to place the ROIs as close as possible to the midpoint of the 5 cm band, when possible, trying to avoid vessels and bile ducts

Using a dedicated workstation (Centricity PACS, RA1000 Workstation and Exam Manager, GE Healthcare), two radiologists independently reviewed all patient cases. For each case, the radiologists independently placed three circular regions of interest (ROIs) in the liver, and three circular ROIs in the spleen on the IOP images. To minimize fluctuations due to coil sensitivity, all ROIs were drawn within a 5 cm deep circular band extending from 10 to 15 cm from the bore isocenter (Fig. 1). ROIs included 100–300 voxels. The three ROIs in the liver were placed on a plane passing through the main portal vein division: one in the right lobe of the liver, one in segment 4 of the liver and one in segment 2/3 of the liver, as demonstratively shown in Fig. 1. All ROIs were placed in the liver avoiding major vessels, ligaments and bile ducts, making sure that each ROI was surrounded by liver parenchyma. ROIs were placed in the same positions on the fat fraction maps and R2* maps. We used the median values for all measured ROIs, in accordance with prior work [29]. Spleen ROIs were placed only on IP and OP images, along the same 5-cm band.

#### Fat fraction, conventional method

The ROIs described above were used to calculate fat fraction with the following “conventional method” formula [12]:

$$ Liver\  fat\  fraction\%=\frac{\frac{SI\ {InPhase}_{liver}}{SI\ {InPhase}_{spleen}}-\frac{SI\ {OutofPhase}_{liver}}{SI\ {OutofPhase}_{spleen}}}{2\ast \frac{SI\ {InPhase}_{liver}}{SI\ {InPhase}_{spleen}}} $$

In three patients, the spleen intensity was markedly decreased, presumably secondary to iron overload. In these patients, ROIs were instead placed in the paraspinal muscles, a less ideal choice given their smaller size and susceptibility to partial volume effects.

#### Fat fraction, IDEAL-IQ method

The liver ROIs described above were placed on the IDEAL-IQ fat fraction reconstruction to generate fat estimates.

#### Iron quantification, conventional method

The multiecho gradient echo acquisition was processed with GE software, which used three liver ROIs to generate T2*/R2* value estimates for each patient. R2* value cutoffs were then employed to assign patients into the following categories: normal liver iron, mildly elevated liver iron, moderately elevated liver iron, severely elevated liver iron. There is a linear correlation between R2* values and liver-iron-concentration (LIC). Since R2* values are directly proportional to iron concentration, they were used as a primary measurement related to intrahepatic iron [29–31]. Increased intrahepatic iron levels were based on an R2* cutoff value that was arbitrary and approximately considered above 71 s^− 1^ based on previous literature, since the only conversion software, approved and commercially available is a propertary software which was not available at the time of the study [30].

### Statistical analysis

Intraclass correlation coefficient (ICC) was estimated to evaluate agreement on iron level and fat fraction between conventional MRI and IDEAL, as well as between two readers. Weighted kappa statistic with squared weights was used to assess agreement between iron level groups (normal: < 71 s^− 1^; mild: 71–270 s^− 1^; moderate: 270–588 s^− 1^; and severe: > 588 s^− 1^) based on conventional MRI measurement and IDEAL measurement as well as between readers [30, 32]. Exact Wilcoxon signed rank test was used to compare iron level and fat fraction between conventional MRI and IDEAL, in all patients, patients with normal iron level, and patients with above normal iron level.

Kappa values were interpreted as follows: 0.00–0.20, slight agreement; 0.21–0.40, fair agreement; 0.41–0.60, moderate agreement; 0.61–0.80, substantial agreement; and 0.81–1.00, almost perfect agreement. A test with *p*-value < 0.05 was considered statistically significant. No adjustment on multiple testing was applied considering hypothesis generating purpose of the study. All statistical analyses were performed in software packages R version 3.3 (The R Foundation for Statistical Computing, Vienna, Austria).

## Results

Three patients were excluded because IDEAL-IQ reconstruction failed due to respiratory motion artifact. One patient was excluded because the T2* mapping GE software failed. The final study population consisted of 50 patients (30 males, 20 females, 50–78 years old).

In the literature, proposed PDFF intervals for each histological steatosis grade in nonalcoholic fatty liver disease are: 0–6.4% for grade 0 (normal); 6.5–17.4% for grade 1 (mild); 17.5–22.1% for grade 2 (moderate); and 22.2% or greater for grade 3 (severe) [19, 33]. Using IDEAL-IQ fat fraction quantification, 12 of 50 patients (26%) had moderate or severe hepatic steatosis and 38 of 50 patients (74%) had normal liver fat or mild hepatic steatosis.

In the literature, 71 s^− 1^ is generally taken as the normal R2* value cutoff [34, 35]. Using IDEAL-IQ quantification, 37 of 50 (74%) patients had abnormal liver iron level, and 13 of 50 (26%) patients had low/normal liver iron levels [30, 32].

**Fig. 2:**
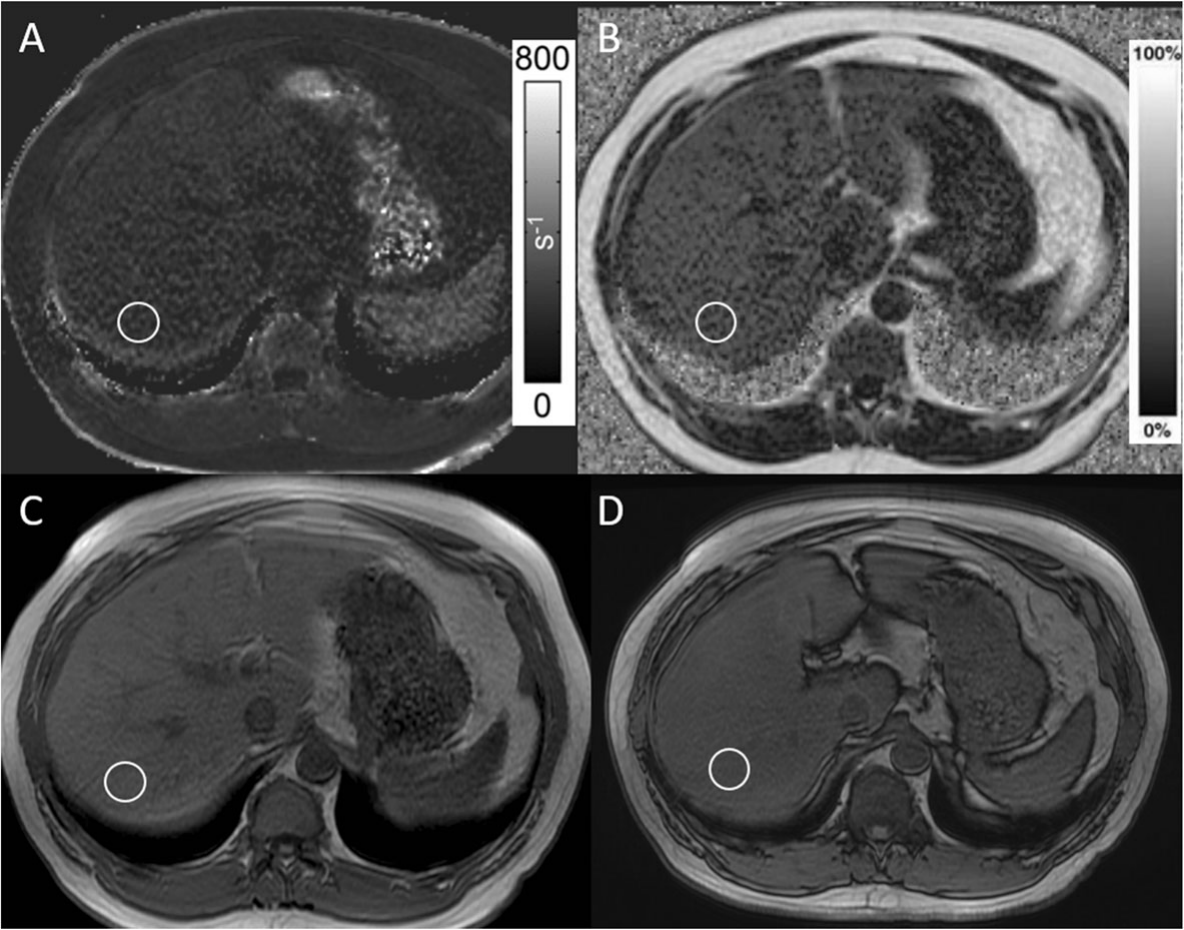
Patient with mild iron overload. **a** R2* map with ROI demonstrating R2* value of 200 s^− 1^. **b** Fat fraction map with ROI demonstrating 25% fat fraction. **c** In-phase and **d** Out-of-phase images demonstrate no signal drop-out within the ROI, demonstrating how elevated liver iron and elevated liver fat cancel on conventional methods and lead to underestimation of both liver fat and liver iron

Ten patients (20%) had both elevated iron and moderate/severe liver fat (Fig. 2). Table 1 summarizes the iron and fat measurements for all methods in both readers.

**Table 1:** Summary of R2* iron measurement and fat fraction in reader 1 and 2

	Median (Range)	Mean ± SD
R2* iron level conventional MRI	109.2 (31.3, 714.3)	126.8 ± 109.6
R2* iron level IDEAL, reader 1	169 (33, 722.2)	194.3 ± 149.5
R2* iron level IDEAL, reader 2	161.8 (32.5, 709.3)	188 ± 140.6
Fat fraction IDEAL, reader 1	11.4 (1, 93.8)	16.3 ± 19.5
Fat fraction IDEAL, reader 2	11.1 (1.2, 99.7)	17.6 ± 21.4
Fat fraction conventional MRI (1), reader 1	1.3 (− 205.5, 35)	−7.4 ± 41.4
Fat fraction conventional MRI (1), reader 2	1.3 (− 148.5, 36.6)	−6.8 ± 35.6
Fat fraction conventional MRI (2), reader 1	1.7 (− 211.6, 34.9)	−6.4 ± 40.6
Fat fraction conventional MRI (2), reader 2	1.8 (− 167.3, 39)	−6.6 ± 37.6

Across all patients, there was almost perfect agreement between readers for IDEAL fat fraction (ICC = 0.957) and IDEAL R2* (ICC = 0.971) measurements, but only substantial agreement between readers for conventional fat fraction measurements (ICC = 0.626). Conventional iron measurements were generated automatically on GE software and thus were not subject to inter-reader agreement analysis.

For both readers, there was poor intra-reader agreement (ICC = 0.026, ICC = 0.028) between conventional fat measurements and IDEAL-IQ fat measurements. For both readers, there was fair intra-reader agreement (ICC = 0.253, ICC = 0.291) between conventional iron measurements and IDEAL-IQ iron measurements (Table 2).

**Table 2:** Agreement on R2* iron level and fat fraction, between conventional MRI and IDEAL, as well as between readers

	ICC	Lower 95%CI	Upper 95%CI
Between conventional MRI and IDEAL			
R2* Iron level, Reader1	0.253	−0.025	0.494
R2* Iron level, Reader2	0.291	0.016	0.524
Fat fraction, Reader1	0.026	−0.252	0.300
Fat fraction, Reader2	0.028	−0.249	0.302
Between readers			
IDEAL R2* iron	0.971	0.950	0.984
IDEAL fat fraction	0.957	0.925	0.975
Conventional fat fraction	0.626	0.422	0.770

Agreement on R2* iron level and fat fraction between conventional MRI and IDEAL was poor. Using IDEAL, the agreement was high between readers on both R2* iron level and fat fraction. On conventional MRI, the reader agreement was moderate on fat fraction

Across all patients, the fat fractions calculated with IOP were statistically significantly different from those calculated with IDEAL-IQ (reader 1: *p* < 0.001, reader 2: *p* < 0.001). When only including patients with above normal iron level (*n* = 37), for both readers, the fat fractions calculated with IOP were statistically significantly different from those calculated with IDEAL-IQ (reader 1: *p* < 0.001, reader 2: *p* < 0.001). However, when only including patients with low/normal iron levels (*n* = 13), the fat fractions calculated with IOP were not clearly statistically significantly different from those calculated with IDEAL-IQ (*p* = 0.068, *p* = 0.048). See Table 3.

**Table 3:** R2* iron level and fat fraction between conventional MRI and IDEAL

	Conventional MRI	IDEAL	*p* Value
All patients			
Fat fraction, reader 1	1.3 (−205.5, 35)	11.4 (1, 93.8)	< 0.001
Fat fraction, reader 2	1.3 (−148.5, 36.6)	11.1 (1.2, 99.7)	< 0.001
Patients with above normal R2* iron level (*n* = 37)			
Fat fraction, reader 1	1.8 (− 121.7, 32)	12.3 (1, 93.8)	< 0.001
Fat fraction, reader 2	0.6 (−148.5, 32.7)	11.9 (1.2, 99.7)	< 0.001
Patients with low/normal R2* iron level (*n* = 13).			
Fat fraction, reader 1	1 (−205.5, 35)	4.5 (1.9, 52.3)	0.068
Fat fraction, reader 2	3.2 (− 115.6, 36.6)	10.3 (2.2, 60)	0.048

## Discussion

In this paper, we demonstrate that a substantial number (20%) of cancer patients undergoing liver MRI at our institution have both elevated liver fat and elevated liver iron. IDEAL-IQ, a confounder-corrected method to measure both liver fat and liver iron, is an important tool for early detection of hepatic steatosis in this patient population.

We show almost perfect inter-reader agreement for both fat measurements using IDEAL-IQ, far better than the conventional methods. This is an important point for clinicians who wish to follow a patient’s hepatic steatosis over time.

Furthermore, we show that there is poor agreement between IOP and IDEAL-IQ fat measurements. We show that the difference is very pronounced in patients with concomitant iron (*p* < 0.001, *p* < 0.001), but not univocally statistically significant in patients with normal levels of iron (*p* = 0.068, *p* = 0.048). This suggests that differences in fat fraction calculation between the IOP and IDEAL-IQ methods are more pronounced at higher iron levels than at lower iron levels. IDEAL-IQ has been previously validated with liver biospies and spectroscopic data and is the gold standard of this study, and so this difference is interpreted as the failure of IOP to measure fat in the presence of iron [36–39]. These results highlight that IOP is an inadequate technique to evaluate for liver fat in cancer patients, who are at high risk of elevated liver iron.

Small sample size did not permit us statistically analyze whether IDEAL-IQ fat fraction measurements were statistically significantly higher than IOP fat measurements. However, as our summary tables show, both the mean and the median of the IDEAL-IQ estimates for both fat and iron were higher than the mean and median conventional method estimates, for all readers. Further evaluation of this trend is warranted, since it suggests that, in the presence of both fat and iron, IDEAL-IQ is more sensitive detector of fat, permitting earlier detection of hepatic steatosis.

The major limitation of this study is the lack of a pathological gold standard; we do not have liver biopsy information for our study population. However, IDEAL-IQ has been pathologically validated in several prior studies [36–39]. As such, it is reasonable to consider IDEAL-IQ the best standard of reference for this study.

## Conclusion

Cancer patients on chemotherapy are at increased risk for concomitantly elevated liver iron and elevated liver fat [4, 6, 40]. We found that 20% of cancer patients undergoing liver MRI with IDEAL-IQ and T2* mapping at our institution demonstrated both elevated liver iron and moderate/severe liver fat. We show that fat measurements using IDEAL-IQ and IOP diverge in patients with concomitantly elevated liver fat and liver iron. Given prior work validating IDEAL-IQ, these diverging measurements indicates that IOP is inadequate to screen for hepatic steatosis in our cancer population IDEAL-IQ demonstrated good inter-reader agreement for all liver iron and liver fat measurements.

## Abbreviations

ICC: Intraclass correlation coefficient
IDEAL-IQ: Iterative decomposition of water and fat with echo asymmetry and least squares estimation
IOP: In and out of phase
IP: In phase
MRI: Magnetic resonance imaging
OP: Out of phase
PDFF: Proton density fat fraction
ROI: Region of interest
SI: Signal intensity
